# Metformin Ameliorates D-Galactose-Induced Senescent Human Bone Marrow-Derived Mesenchymal Stem Cells by Enhancing Autophagy

**DOI:** 10.1155/2023/1429642

**Published:** 2023-03-30

**Authors:** Pingting Ye, Lei Feng, Dan Zhang, Ruihao Li, Yixuan Wen, Xiaohan Tong, Shuo Shi, Chunyan Dong

**Affiliations:** Department of Oncology, Shanghai East Hospital, School of Medicine, Shanghai Key Laboratory of Chemical Assessment and Sustainability, School of Chemical Science and Engineering, Tongji University, Shanghai 200120, China

## Abstract

Human bone marrow-derived mesenchymal stem cells (hBMSCs) are promising candidates for stem cell therapy in clinical trials. Applications of hBMSCs in clinical therapy are limited by cellular senescence due to long-term *ex vivo* expansion. Metformin, an oral hypoglycemic drug for type 2 diabetes, has been shown to have antiaging effects. However, the mechanisms of metformin in antiaging treatment remain controversial. Here, we used D-galactose (D-gal) to establish an appropriate model of senescent hBMSCs to explore the antiaging effects of metformin. Following metformin treatment with a low concentration range, senescence phenotypes induced by D-gal significantly changed, including generation of reactive oxygen species (ROS), loss of mitochondrial membrane potential (MMP), and cell cycle arrest. In contrast, no apparent change was found in unsenescent hBMSCs. Furthermore, the results show that activation of 5′AMP-activated protein kinase (AMPK) by metformin enhances cell autophagy in senescent hBMSCs. These findings suggest that metformin exerts antiaging function within the low concentration range by enhancing autophagy and exhibits potential benefits for clinical stem cell therapy by ameliorating the *ex vivo* replicative senescence of hBMSCs.

## 1. Introduction

Mesenchymal stem cells (MSCs) possess the abilities of self-renewal and multidirectional differentiation, which can be isolated from various human tissues (such as bone marrow, umbilical, and adipose) [1]. Additionally, human bone marrow-derived MSCs (hBMSCs) are candidates for clinical stem cell therapy due to their low immunogenicity and easy *in vitro* expansion. So far, more than 460 clinical trials of hBMSCs have been registered in the database (https://clinicaltrials.gov/). Though current *in vitro* expansion technology is mature, replicative senescence is inevitable, which critically limits the application of hBMSCs in therapy [2, 3].

Cellular senescence, a state of stable and irreversible growth arrest, is characterized by macromolecular damage and metabolic, morphological, and functional alterations [4, 5]. D-Galactose (D-gal), a physiological reducing sugar, is widely applied for constructing cell and animal aging models [6–8]. The oversupply of D-gal can be used to model the natural or replicative senescence of cells. In the present study, an appropriate dose of D-gal was used to induce senescence of hBMSCs *in vitro* without cell-killing effects.

Metformin is an oral hypoglycemic drug and has been applied in first-line treatment for type 2 diabetes worldwide [9]. In the early 2000s, it has been reported that metformin can decelerate aging and increase lifespan in mice [10, 11]. Afterwards, various studies have shown that metformin involves in regulating the hallmarks of aging in multiple model organisms, including *C. elegans*, *Drosophila*, rodents, and human cell lines [12]. Moreover, under the funding from the American Federation of Aging Research, a large clinical trial “Targeting Aging by Metformin” (TAME) was designed to test the age-targeting effects of metformin [13, 14].

Metformin involves multiple pathways to target aging; however, the mechanisms are complex [15, 16]. Especially, metformin exerts biphasic effects in mitochondria depending on cell types, drug duration, and concentration [17, 18]. A previous study has shown that pharmacological metformin concentrations increase mitochondrial activities via activating 5′AMP-activated protein kinase (AMPK) signaling pathway, whereas suprapharmacological metformin concentrations inhibit mitochondrial activities by reducing the level of adenine nucleotides [19]. Nevertheless, the results were obtained from a model of normal primary hepatocytes, but not senescent cells.

Senescent cells exhibit mitochondrial dysfunction, which reflects in the changes of dynamics, morphology, and function [4]. The dysfunction of mitochondria can induce more reactive oxygen species (ROS) accumulation, which contributes to aggravating cell senescence [20]. Recently, Bharath et al. showed that pharmacological metformin concentration (100 *μ*M) can enhance mitochondrial function in senescent T cells [21]. However, the involvement of metformin in modulating mitochondrial function of senescent hBMSCs is still unknown. Bharath et al. also reported that metformin enhanced senescent T cell autophagy *in vitro* [21]. Autophagy serves as an essential role in cellular homeostasis. Based on previous studies, it appears that the association of autophagy and senescent MSCs is complex and controversial [22, 23].

In the present study, an appropriate model of senescent hBMSCs was established for exploring the antiaging effects of metformin *in vitro*. Data herein show that the low concentration range of metformin can ameliorate senescence phenotypes of hBMSCs, including generation of ROS, loss of mitochondrial membrane potential (MMP), and cell cycle arrest. Metformin has more significant impacts on senescent hBMSCs compared to unsenescent hBMSCs. Moreover, metformin reduces senescence of hBMSCs by enhancing autophagy mediated by the AMPK pathway. It concludes that metformin exerts antiaging function within the low concentration range by enhancing autophagy. And metformin exhibits a potential benefit for clinical stem cell therapy by ameliorating *ex vivo* replicative senescence of hBMSCs.

## 2. Materials and Methods

### 2.1. Reagents and Antibodies

Metformin and compound C (CoC) were purchased from MCE; D-gal was purchased from Sigma-Aldrich; anti-p21^Waf1/Cip1^ (2947), anti-p53 (2527), anti-AMPK*α* (5831), and anti-phospho-AMPK*α* (Thr172) (2535) were purchased from Cell Signaling Technology; anti-LC3B (T55992) was obtained from Abmart; anti-CDKN2A/p16^INK4a^ (ab108349) was purchased from Abcam; GAPDH (60004-1-Ig) was obtained from Proteintech.

### 2.2. Cell Culture and Treatment

hBMSCs were cultured in DMEM containing 10% FBS and 1% penicillin-streptomycin at 37°C in a humidified incubator of 5% CO_2_. To ensure the consistency, the same generations of hBMSCs were used in the experiments. Before any treatments, hBMSCs were cultured in the normal medium for 24 h.

### 2.3. Cell Viability Assay

Cell Counting Kit 8 (CCK-8; KeyGEN BioTECH) was used to measure cell viability. For cytotoxicity assay, cells were seeded in a 96-well plate at 1 × 10^4^/well and treated with 0-100 g/L D-gal for 24 h; for cell proliferation assay, cells were seeded in a 96-well plate at 1 × 10^3^/well and treated with 100 *μ*L complete medium, while the 20 g/L D-gal+metformin groups with 0, 10, 50, 100, 200, and 2000 *μ*M metformin. After incubation with 10 *μ*L CCK8 solution for 2 h, the absorbance of each well was measured at 450 nm.

### 2.4. Senescence Associated *β*-Galactosidase (SA-*β*-Gal) Staining

SA-*β*-gal staining kit (Beyotime, C0602) was used to measure cell SA-*β*-Gal activity. Briefly, the cells were washed with phosphate-buffered saline (PBS) and incubated in a fixative solution for 15 minutes at room temperature. Afterwards, the cells were washed with PBS and incubated in SA-*β*-Gal staining solution at 37°C overnight without CO_2_. The images were captured under an inverted microscope, and the positive cells were quantified from 5 fields of each well.

### 2.5. Detection of Intracellular ROS

ROS assay kit (Beyotime, S0033) was used to detect intracellular ROS production. Briefly, the cells were collected and incubated with 10 *μ*M 2′,7′-dichlorofluorescein diacetate (DCFH-DA) at 37°C in the dark for 20 minutes. After being washed with serum-free medium three times, the fluorescence intensity was quantified by a flow cytometer (BD FACSAria™ II Cell Sorter).

### 2.6. Detection of MMP

MMP assay kit (Beyotime, C2006) was used to evaluate the MMP. Briefly, the cells were collected and incubated with JC-1 staining solution at 37°C for 20 minutes. After being washed with JC-1 buffer twice, the fluorescence intensity was quantified by a flow cytometer (BD FACSAria™ II Cell Sorter). MMP can be evaluated based on red fluorescence (JC-1 aggregates)/green fluorescence (JC-1 monomers) intensity ratio.

### 2.7. Cell Cycle Assay

Cell cycle assay (KeyGEN BioTECH, KGA512) was used to analyze the distribution of cell cycle. Briefly, the cells were collected and fixed with 70% ethanol at 4°C overnight. Afterwards, the cells were washed with PBS and incubated in propidium iodide (PI)/RNase A staining solution at room temperature in the dark for 30 minutes. The distribution of cell cycle was detected by a flow cytometer (BD FACSAria™ II Cell Sorter).

### 2.8. Western Blot Analysis

The cells were washed with ice-cold PBS twice and lysed in ice-cold RIPA buffer (Beyotime, P0013B) containing 1% PMSF (Beyotime, ST506) and 1% protease inhibitor cocktail (MCE, HY-K0010) for 15 minutes. After centrifugation (1.3 × 10^4^ rpm, 30 minutes) at 4°C, total protein concentrations of the supernatant were quantified by BCA protein assay kit (Beyotime, P0010). The standard protein samples (40 *μ*g) were separated by SDS-PAGE and then transferred to polyvinylidene difluoride (PVDF) membranes. After being blocked with 5% skimmed milk, the membranes were incubated with primary antibodies overnight at 4°C. Subsequently, the membranes were washed with TBST (Beyotime, ST673) and incubated with secondary antibody for 2 hours at room temperature. After being washed with TBST three times, the protein blots were visualized by enhanced chemiluminescence and autoradiography.

### 2.9. Statistical Analysis

All results were represented as mean ± SEM. The GraphPad Prism version 8.0 (GraphPad Software, CA, USA) was used to analyze data. Statistical analyses among different groups were performed by ANOVA and Student *t* test. Statistically significant differences were indicated as ^∗^*p* < 0.05, ^∗∗^*p* < 0.01, ^∗∗∗^*p* < 0.001, and ^∗∗∗∗^*p* < 0.0001.

## 3. Results

### 3.1. D-gal Induced hBMSC Senescence in a Concentration-Dependent Manner

**Figure 1:**
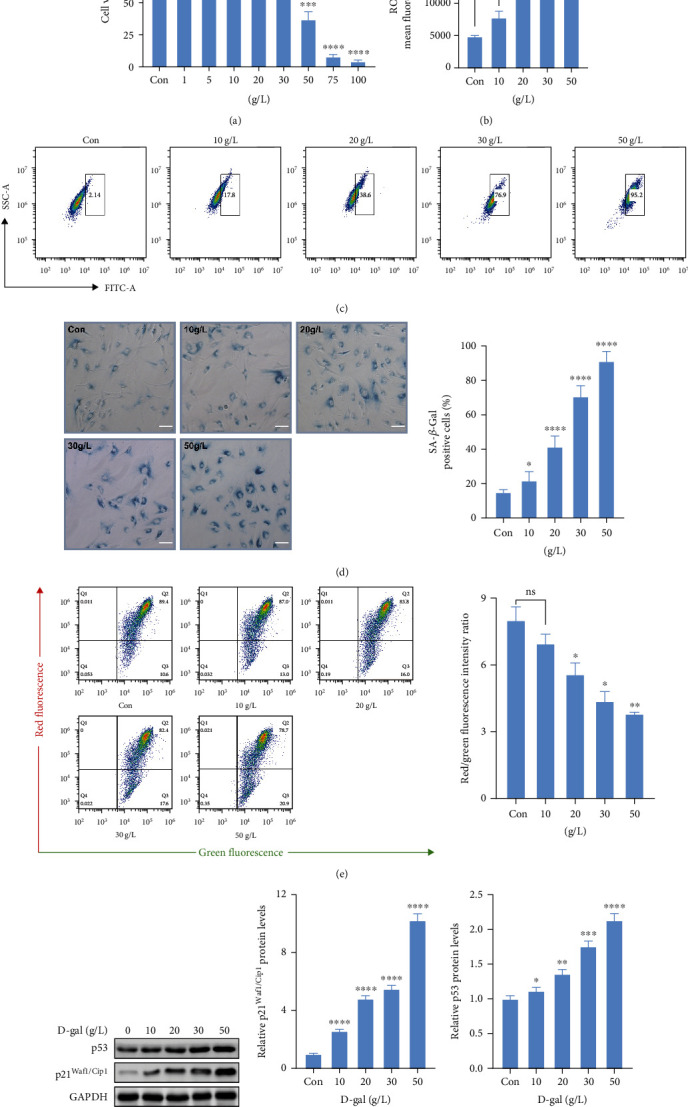
The cytotoxicity of D-gal on hBMSCs and effects of different concentrations D-gal on hBMSC senescence. (a) The cell viability of hBMSCs was assessed by CCK8 assay following treatment with different concentrations of D-gal (0-100 g/L) for 24 h. (b) Quantification of intracellular ROS levels by flow cytometry. (c) Representative images of flow cytometry of intracellular ROS levels. (d) Representative images of SA-*β*-Gal-positive hBMSCs, scale bar, 100 *μ*m. (e) Quantification of MMP by flow cytometry. (f–h) The expressions of p21^Waf1/Cip1^ and p53 proteins were detected by western blot analysis. GAPDH was used as a control. *n* = 2 − 3 for each group. Data shown are mean ± SEM. Two-tailed *t* test. ^∗^*p* < 0.05, ^∗∗^*p* < 0.01, ^∗∗∗^*p* < 0.001, and ^∗∗∗∗^*p* < 0.0001 versus the control group; ns: not significant.

First, we assessed the cytotoxicity of D-gal on hBMSCs by CCK8 assay. After being treated with different concentrations of D-gal (0-100 g/L) for 12 h, the cell viability showed no change compared with the control group at the concentration of 25 g/L (Supplementary Figure S1). However, the result showed 13.19% inhibition of cell viability at the concentration of 20 g/L of D-gal following 24 h treatment and revealed a significant difference (*p* = 0.0499) compared with the control group (Figure 1(a)). Considering the effect of D-gal on arresting cell proliferation, the results indicated that low-dose D-gal could induce senescence in hBMSCs without cell cytotoxicity.

Given the generation of intracellular ROS is a typical feature of senescent cells [24, 25], a significant increase of ROS DCFH mean fluorescence intensity (MFI) was found in a concentration-dependent manner (ranged from 0 to 50 g/L) by flow cytometry (Figures 1(b) and 1(c) and Supplementary Figure S2). SA-*β*-Gal staining, as a widely used method for cell senescence assessment, was performed to reveal the effect of D-gal on hBMSC senescence [26]. Compared to the control group (15.20 ± 0.58%), only 10 g/L D-gal (22.00 ± 2.24%) could significantly increase the number of SA-*β*-Gal-positive cells (Figure 1(d)).

To explore the effect of D-gal on mitochondrial function, the MMP levels of hBMSCs were measured by flow cytometry. Quantification of MMP is presented by red fluorescence (JC-1 aggregates)/green fluorescence (JC-1 monomers) intensity ratio. As shown in Figure 1(e), the ratio of red/green fluorescence intensity decreases significantly with increasing concentrations of D-gal. The results revealed that D-gal could induce MMP loss in a concentration-dependent manner. Additionally, the cellular senescence markers of hBMSCs including p21^Waf1/Cip1^ and p53 were clearly increased with increasing concentrations of D-gal (Figures 1(f)–1(h)).

Taken together, these data demonstrated that D-gal induced senescence and mitochondrial dysfunction in hBMSCs in a concentration-dependent manner. To rule out the effect of D-gal on cell cytotoxicity, the optimal concentration of 20 g/L of D-gal was chosen for subsequent experiments.

### 3.2. Metformin Inhibited Senescence of D-gal-Induced hBMSCs

**Figure 2:**
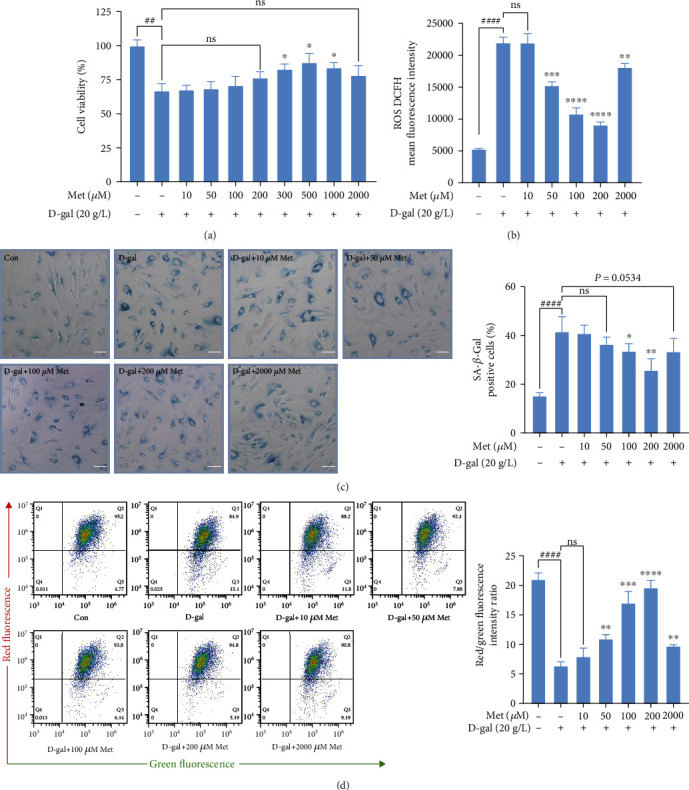
The effects of metformin on improving senescence phenotypes of D-gal-induced hBMSCs. (a) The cell viability of hBMSCs was assessed by CCK8 assay after cultured for 72 h. (b) Quantification of intracellular ROS levels by flow cytometry. (c) Representative images of SA-*β*-Gal-positive hBMSCs, scale bar, 100 *μ*m. (d) Quantification of MMP by flow cytometry. *n* = 3 for each group. Data shown are mean ± SEM. Two-tailed *t* test. ^∗^*p* < 0.05, ^∗∗^*p* < 0.01, ^∗∗∗^*p* < 0.001, and ^∗∗∗∗^*p* < 0.0001 versus the D-gal group; ^##^*p* < 0.01 and ^####^*p* < 0.0001 versus the control group; ns: not significant.

To evaluate the effects of different metformin concentrations on senescence of 20 g/L D-gal-induced hBMSCs, we assessed cell proliferation by CCK8 assay (Figure 2(a)). Within the low concentration range (10-500 *μ*M) of metformin for 72 h, cell viability of senescent hBMSCs was restored in a concentration-dependent relationship. Note that although the 20 g/L D-gal+1 mM metformin and 20 g/L D-gal+2 mM metformin groups were able to significantly restore cell proliferation compared to the 20 g/L D-gal group, the ability showed a tendency to decline. After being treated with 50 g/L D-gal, similar results were found (Supplementary Figure S3).

ROS DCFH MFI in the D-gal+200 *μ*M metformin group was less than half (41.45%) in the D-gal group. Compared with the D-gal group, intracellular ROS levels were clearly decreased in the D-gal+metformin groups by measuring DCFH MFI (Figure 2(b) and Supplementary Figure S2). The cell count revealed that the percentage of SA-*β*-Gal-positive cells was obviously decreased after incubation with low-dose metformin (10-200 *μ*M), while no significant difference was found between the D-gal+2 mM metformin group and D-gal group (*p* = 0.0534) (Figure 2(c)). Next, MMP levels were measured using a flow cytometry-based JC-1 staining. Consistent with the results of cell proliferation assay, high-dose metformin (2 mM) could improve senescence phenotypes of D-gal-induced hBMSCs, while the ability of it was weaker than low-dose metformin (10-200 *μ*M). However, the D-gal+metformin groups showed higher MMP levels than the D-gal group in general (Figure 2(d)).

Taken together, these findings suggested that metformin exerted an antiaging function in senescent hBMSCs within the low concentration range and the opposite effect within the high concentration range.

### 3.3. Metformin Reversed Cell Cycle Arrest in Senescent hBMSCs

**Figure 3:**
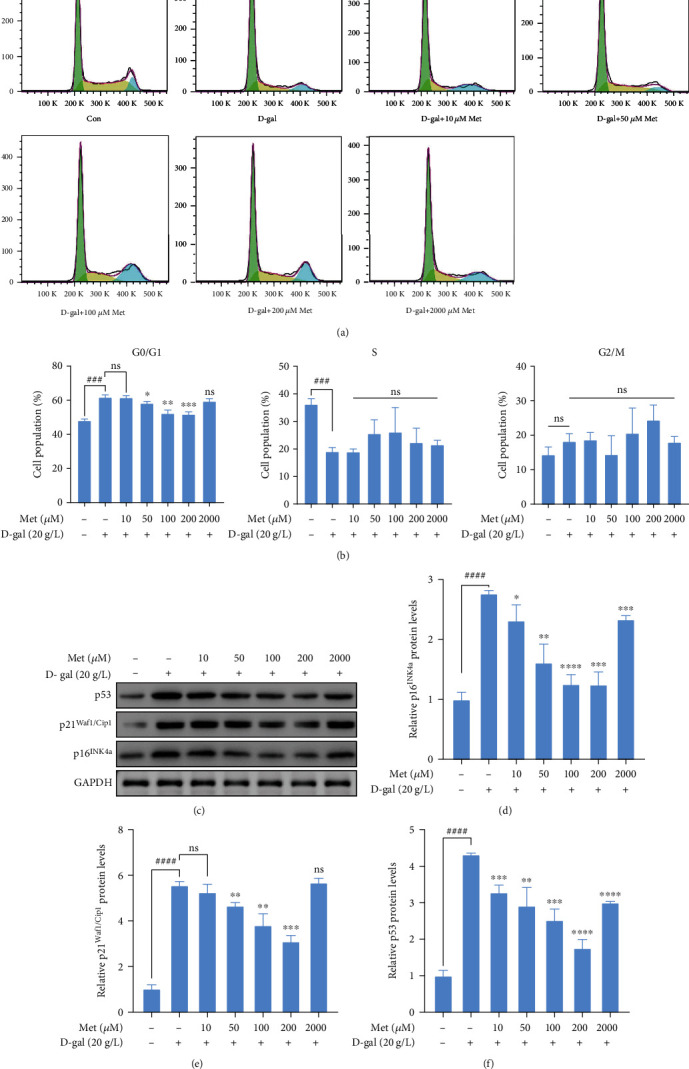
The effect of metformin on reversing cell cycle arrest in D-gal-induced hBMSCs. (a) Analysis of cell cycle distribution by flow cytometry. (b) Bar graph of cell cycle distribution. (c–f) The expressions of p16^INK4a^, p21^Waf1/Cip1^, and p53 proteins were detected by western blot analysis. GAPDH was used as a control. *n* = 3 for each group. Data shown are mean ± SEM. Two-tailed *t* test. ^∗^*p* < 0.05, ^∗∗^*p* < 0.01, ^∗∗∗^*p* < 0.001, and ^∗∗∗∗^*p* < 0.0001 versus the D-gal group; ^###^*p* < 0.001 and ^####^*p* < 0.0001 versus the control group; ns: not significant.

Cellular senescence is characterized by the arrest of the cell cycle. To investigate the effect of metformin on cell cycle distribution of D-gal-induced hBMSCs, cell cycle analysis was performed by flow cytometry (Figures 3(a) and 3(b)). The D-gal group (61.76 ± 0.82%) had a significantly higher proportion of hBMSCs in G0/G1 phase than the control group (48.11 ± 0.51%, *p* < 0.001), while the proportion of hBMSCs in S phase was significantly lower (*p* < 0.001). After incubation with metformin for 48 h, the D-gal-induced hBMSCs in G0/G1 phase gradually decreased in a concentration-dependent manner (10-200 *μ*M). In mammalian cells, cyclin-dependent kinases (CDKs) and p53 proteins are key factors for regulating cell cycle [27, 28]. p16^INK4a^ (a CDK4/6 inhibitor), p21^Waf1/Cip1^ (a CDK2 inhibitor), and p53 proteins are canonical senescence markers [4, 29]. To investigate the expression of these proteins, western blot analysis was performed (Figure 3(c)). The expressions of p16^INK4a^, p21^Waf1/Cip1^, and p53 proteins were obviously increased in the D-gal group (*p* < 0.0001), whereas the D-gal+metformin (10-200 *μ*M) groups showed a clear decrease in these protein expressions compared with the D-gal group (Figures 3(d)–3(f)).

Taken together, these data demonstrated that D-gal induced cell cycle arrest of hBMSCs and low-dose metformin reversed cell cycle arrest in senescent hBMSCs.

### 3.4. Metformin Improved the Impaired Autophagic Flux in D-gal-Induced hBMSCs by AMPK Activation

**Figure 4:**
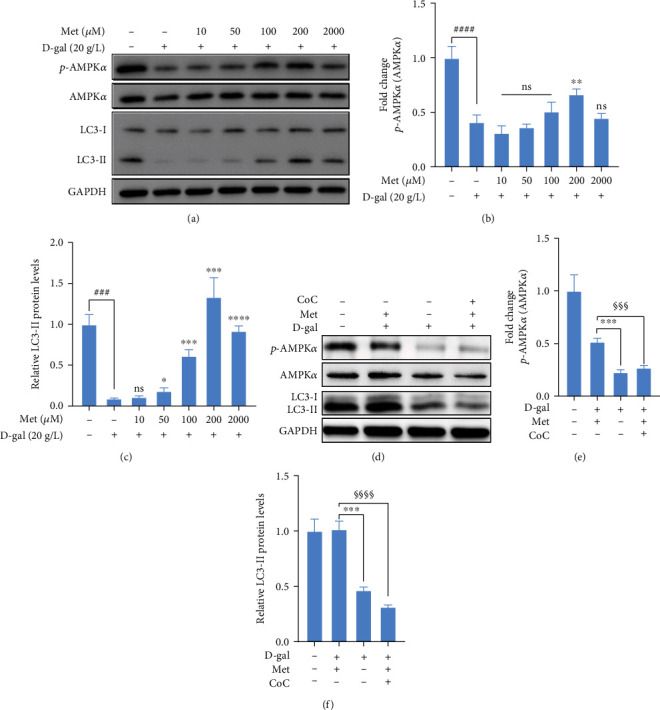
Metformin regulated autophagic flux in D-gal-induced hBMSCs by AMPK pathway. (a–c) Western blot analysis of AMPK*α*, p-AMPK*α*, and LC3 proteins in hBMSCs treated or not with D-gal or metformin. GAPDH was used as a control. (d–f) Western blot analysis of AMPK*α*, p-AMPK*α*, and LC3 proteins in hBMSCs treated or not with 20 g/L D-gal or 200 *μ*M metformin or 5 *μ*M CoC. GAPDH was used as a control. *n* = 3 for each group. Data shown are mean ± SEM. Two-tailed *t* test. ^∗^*p* < 0.05, ^∗∗^*p* < 0.01, ^∗∗∗^*p* < 0.001, and ^∗∗∗∗^*p* < 0.0001 versus the D-gal group; ^###^*p* < 0.001 and ^####^*p* < 0.0001 versus the control group; ^§§§^*p* < 0.001 and ^§§§§^*p* < 0.0001 versus the D-gal+metformin+CoC group; ns: not significant.

As for hBMSCs, autophagy is critical for cellular homeostasis [23]. To explore the role of metformin in modulating autophagy in D-gal-induced hBMSCs, the expressions of AMPK*α*, p-AMPK*α*, and LC3 proteins were detected by western blot analysis (Figure 4(a)). The ratio of p-AMPK*α*/AMPK*α* protein expressions was significantly higher in the D-gal+200 *μ*M metformin group than the D-gal group (*p* < 0.01). Compared with the D-gal+metformin (10-100 *μ*M) groups and D-gal group, although the difference was not statistically significant, an obvious trend was shown (Figure 4(b)). The results showed that metformin activated AMPK pathway in a concentration-dependent manner (10-200 *μ*M). The expression of LC3-II protein was more obvious in the D-gal+metformin groups compared with the D-gal groups (Figure 4(c)). The enhanced LC3-I to LC3-II conversion rate indicated that metformin improved autophagy in D-gal-induced hBMSCs.

To further verify that metformin regulated autophagic flux in D-gal-induced hBMSCs via regulating the AMPK pathway, AMPK inhibitor (CoC) was used to examine the effects of metformin (Figures 4(d)–4(f)). The results showed that inhibition of AMPK with CoC lowered the ratio of p-AMPK*α*/AMPK*α* protein expressions and the accumulation of LC3-II protein compared with the D-gal+200 *μ*M metformin group. Coadministration of 5 *μ*M CoC completely abolished the effects of metformin on improving the impaired autophagic flux.

Taken together, these findings suggested that metformin enhanced autophagy to exert antiaging function by activating the AMPK pathway.

### 3.5. Metformin Played a Minor Role in Unsenescent hBMSCs but Had a Significant Impact on Senescent hBMSCs

**Figure 5:**
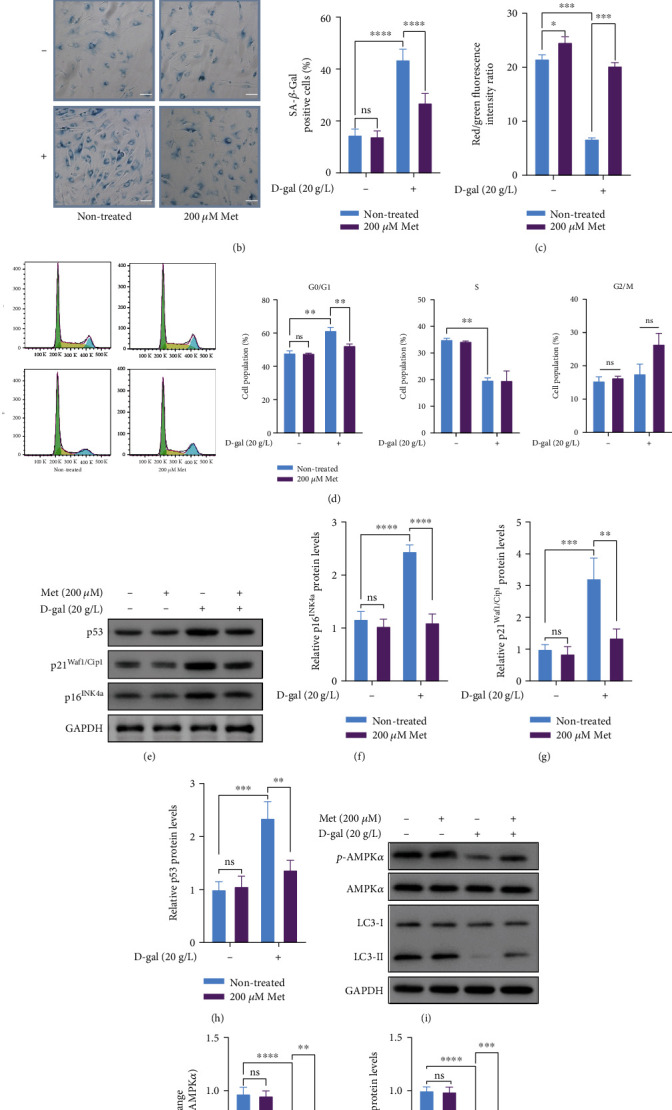
Metformin significantly ameliorated D-gal-induced senescent hBMSCs, but not unsenescent hBMSCs. (a) Quantification of intracellular ROS levels by flow cytometry. (b) Representative images of SA-*β*-Gal-positive hBMSCs, scale bar, 100 *μ*m. (c) Quantification of MMP by flow cytometry. (d) Analysis of cell cycle distribution by flow cytometry. (e–h) The expressions of p16^INK4a^, p21^Waf1/Cip1^, and p53 proteins were detected by western blot analysis. GAPDH was used as a control. (i–k) The expressions of AMPK*α*, p-AMPK*α*, and LC3 proteins were detected by western blot analysis. GAPDH was used as a control. *n* = 3 for each group. Data shown are mean ± SEM. Analysis of variance (ANOVA). ^∗^*p* < 0.05, ^∗∗^*p* < 0.01, ^∗∗∗^*p* < 0.001, and ^∗∗∗∗^*p* < 0.0001 versus the D-gal group; ns: not significant.

To explore the antiaging function of metformin in unsenescent hBMSCs, senescence phenotypes of hBMSCs were examined. After incubation with 200 *μ*M metformin for 48 h, intracellular ROS levels did no change significantly in unsenescent hBMSCs (*p* > 0.05), while the decline for the D-gal+metformin group was 58.7% compared with the D-gal group (*p* < 0.0001, Figure 5(a) and Supplementary Figure S2). After SA-*β*-Gal staining, no obvious change was found in unsenescent hBMSCs following treatment of metformin (Figure 5(b)). MMP levels were significantly changed in both unsenescent and senescent hBMSCs (Figure 5(c) and Supplementary Figure S4). The magnitude of changes was much greater in the senescent hBMSCs than in the unsenescent hBMSCs. To investigate the effect of metformin on cell cycle arrest between unsenescent and senescent hBMSCs, the result of cell cycle analysis revealed that the metformin group (47.79 ± 0.11%) and nontreated group (48.09 ± 0.89%) had about the same proportion of hBMSCs in G0/G1 phase (Figure 5(d)). Besides, there were no obvious differences observed for expression of senescence-related proteins in unsenescent hBMSCs (p16^INK4a^, p21^Waf1/Cip1^, and p53, Figures 5(e)–5(h)). Consistently, metformin had no significant impact on the expression of p-AMPK*α* and LC3-II proteins in unsenescent hBMSCs (Figures 5(i)–5(k)). Taken together, these findings demonstrated that metformin exerts obvious antiaging function in senescent hBMSCs but has less significant impact on unsenescent hBMSCs.

## 4. Discussion

It is currently recognized that MSCs have potent antiproliferative and anti-inflammatory effects. Therefore, MSCs have been widely used in clinical stem cell therapy to treat traumatic and degenerative disorders [30–32]. For practical applications, a sufficient number of MSCs are required via *in vitro* expansion. However, cellular senescence is inevitable during *in vitro* expansion, which greatly influences therapeutic efficacy [33]. Recently, numerous studies have revealed the mechanisms and therapeutic strategies to ameliorate cellular senescence [34–36]. Although the antiaging function of metformin has been delineated in many cell and animal models, the regulation pathway of metformin in senescent hBMSCs remains unknown [21, 37, 38]. In the present study, we use D-gal to model senescent hBMSCs and demonstrate that metformin ameliorates *ex vivo* senescence in hBMSCs by enhancing cell autophagy within the low concentration range.

D-gal has been widely used to model aging; however, there is no uniform standard for optimal dosage. It could be caused by different sources of D-gal and different cell sensitivities. Similar to the findings of Xu et al. [8], our results revealed that D-gal induced cellular senescence in a time- and concentration-dependent manner. Considering the cytotoxicity of high-dose D-gal on hBMSCs, we chose 20 g/L D-gal as the optimal concentration in this study. A previous study has explored hBMSC aging via *in vitro* passage [3]. However, due to the highly differentiated character of hBMSCs, cellular differentiation occurred with the number of passages. In our study, using D-gal to induce cell senescence was successful in avoiding cell differentiation and ensuring cell homogeneity in the same experiment.

Metformin, as an oral hypoglycemic drug, possesses a high safety profile and has been shown a significant correlation with aging [12]. According to previous studies, metformin was used at a wide range of concentrations (10 *μ*M to 10 mM) to ameliorate senescence [21, 37, 39, 40]. Our data showed that 500 *μ*M metformin exerted the best antiaging effect in hBMSCs, while excessive metformin concentrations might have opposite impact. The differences may be caused by different cell types and treatment times. Kim et al. suggested that merely 10 *μ*M metformin had effective antiaging function in adipose tissue-derived MSCs [37]. Although it has been shown that different sources of MSCs have no significant differences [41], hBMSCs in our study have no obvious changes, when treated with 10 *μ*M metformin for 48 h. Additional research is needed to elaborate on this distinction. For clinical application, the optimal therapeutic dose of metformin is 2 g/day, and metformin concentrations are around 40-80 *μ*M in the portal vein [42]. Our findings demonstrated that pharmacological metformin concentrations had antiaging effects on senescent hBMSCs, including reduced ROS and increased MMP levels. Consistently, pharmacological metformin concentration ameliorates T cell senescence to alleviate aging-associated inflammation [21]. Based on existing findings, metformin is a potential future age-targeting drug.

Mitochondria, as the center of cell energy metabolism, are highly associated with cellular senescence. Wang et al. and Bharath et al. all reported that metformin could increase MMP levels at pharmacological concentration [19, 21]. In this study, we also found higher MMP levels following treatment with metformin. Consistent with the study by Wang et al. [19], our results revealed that a low concentration range of metformin treatment improved MMP levels of senescent hBMSCs in a concentration-dependent manner, while high concentration range of metformin treatment reduced MMP levels. Autophagy has been identified as a crucial regulation role in energy metabolism [43, 44], which is closely related to mitochondrial function [21, 45]. Autophagy serves as a senescent hallmarks and an essential catabolic process for cellular homeostasis under normal and stress conditions [22]. However, the effect of autophagy on hBMSC aging is complex. In this study, we observed that D-gal induced loss of autophagy and metformin reversed it. The results are contrary to a previous study, which revealed that high glucose promotes autophagy to induce senescence in hBMSCs [46].

Cellular senescence is a chronic process that occurs due to the accumulation of low intensity stress. Under normal conditions, autophagy sustains a low level which is mainly for maintaining cellular homeostasis [47]. But, under stressed conditions, autophagy is activated for maintaining stemness of stem cells [48, 49]. As aging progresses, aged stem cells exhibit impaired autophagy, which results in stemness loss and metabolic disorder. According to our results, autophagic flux of hBMSCs was impaired under D-gal treatment. But the impaired autophagic flux was improved following the treatment with metformin. Therefore, it was concluded that metformin can reverse senescence of hBMSCs via enhancing autophagy. Moreover, metformin played a minor role in unsenescent hBMSCs as compared to senescent hBMSCs. The results were similar to a recent study [21]. Additionally, a clinical trial was designed to explore antiaging and proautophagy effects of metformin in adults with prediabetes (NCT03309007). According to the above data, metformin might have antiaging potential by targeting autophagy. However, the precise role and molecular mechanisms involved in this process remain to be elucidated.

One limitation of our study is that we only examined the antiaging effect of metformin in D-gal-induced hBMSC senescence, but not in replication-induced hBMSC senescence. More experiments will be conducted to compare the differences between the two types of senescence. However, as we discussed above, differentiation of hBMSCs is inevitable with the number of passages. Therefore, how to maintain cell homogeneity remains to be addressed. Another limitation is that this study was conducted only in an *ex vivo* model. For practical applications, whether therapeutic doses of metformin can achieve effective concentrations in bone marrow remains to be explored. Future *in vivo* experiments in human need to be designed elaborately.

## 5. Conclusions

**Figure 6:**
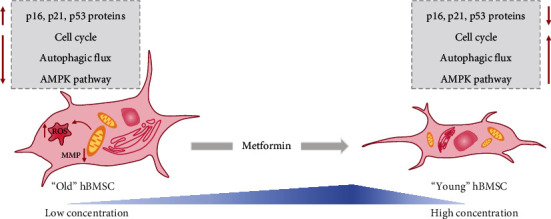
Schematic diagram of antiaging effect of metformin in hBMSCs. Metformin exerts antiaging function within the low concentration range by enhancing autophagic flux. hBMSCs: human bone marrow-derived mesenchymal stem cells; ROS: reactive oxygen species; MMP: mitochondrial membrane potential; AMPK: 5′AMP-activated protein kinase.

Our study demonstrates that the low concentration range of metformin has antiaging impact on senescent hBMSCs by enhancing autophagy (Figure 6). The findings showed clinical application prospect of metformin in stem cell therapy. Further experiments to evaluate the efficacy and feasibility of this application are necessary in the future.

## Data Availability

The data used to support the findings of this study are available from the corresponding authors upon request.

## Supplementary Materials

Supplementary Materials Supplementary Figure S1: the cell viability of hBMSCs was assessed by CCK8 assay following treatment with different concentrations of D-gal (0-100 g/L) for 12 h. *n* = 3 for each group. Data shown are mean ± SEM. Two-tailed *t* test. ^∗^*p* < 0.05, ^∗∗∗^*p* < 0.001, and ^∗∗∗∗^*p* < 0.0001 versus the control group; ns: not significant. Supplementary Figure S2: flow cytometry analysis of intracellular ROS levels. *n* = 3 for each group. Supplementary Figure S3: the cell viability of hBMSCs was assessed by CCK8 assay after being cultured for 72 h (at the concentration of 50 g/L D-gal). *n* = 3 for each group. Data shown are mean ± SEM. Two-tailed *t* test. ^∗^*p* < 0.05, ^∗∗^*p* < 0.01, and ^∗∗∗^*p* < 0.001 versus the D-gal group; ^####^*p* < 0.0001 versus the control group; ns: not significant. Supplementary Figure S4: flow cytometry analysis of MMP. *n* = 3 for each group.
