# Supplementary material for: Metformin Ameliorates D-Galactose-Induced Senescent Human Bone Marrow-Derived Mesenchymal Stem Cells by Enhancing Autophagy
Source: Stem Cells Int. 2023 Mar 30;2023:1429642. doi: 10.1155/2023/1429642 (PMC10079386; doi:10.1155/2023/1429642)

**Supplementary Figure S1**


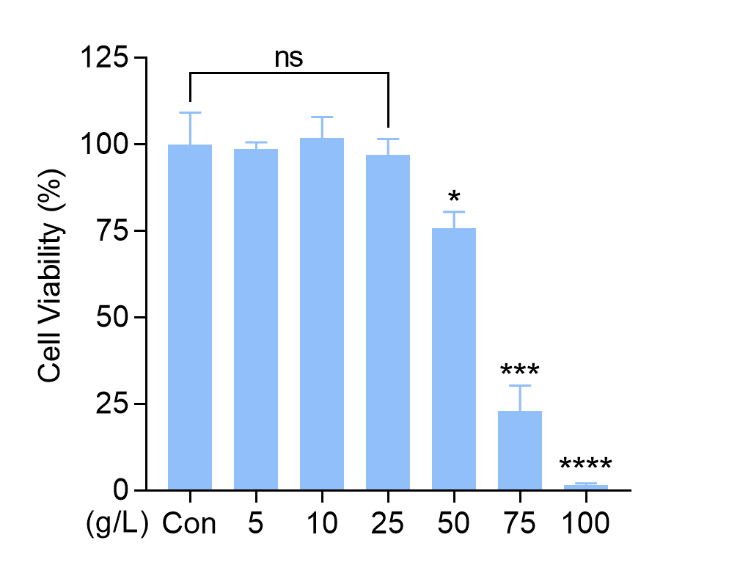


**Supplementary Figure S2**

**
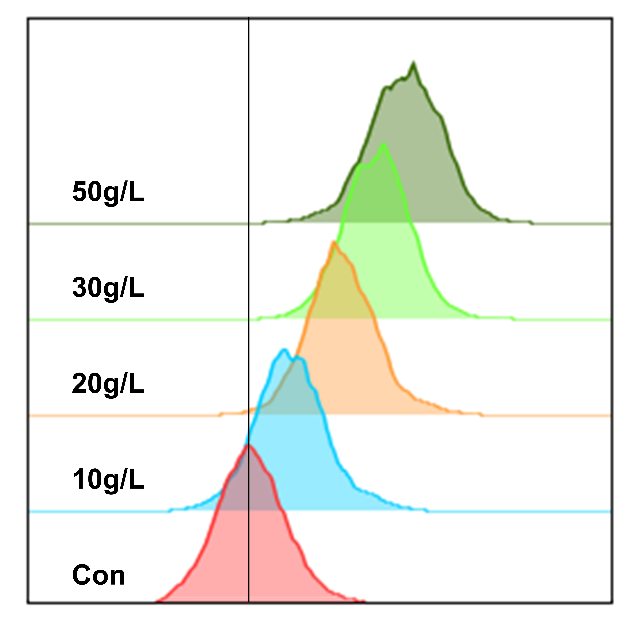
**


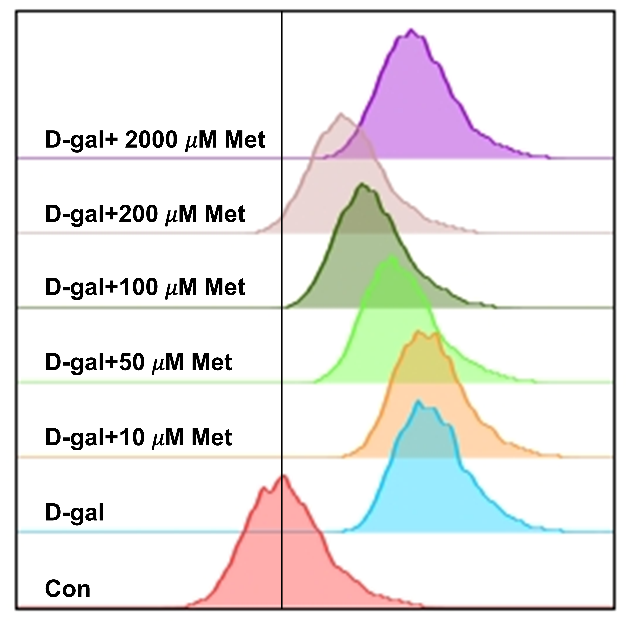


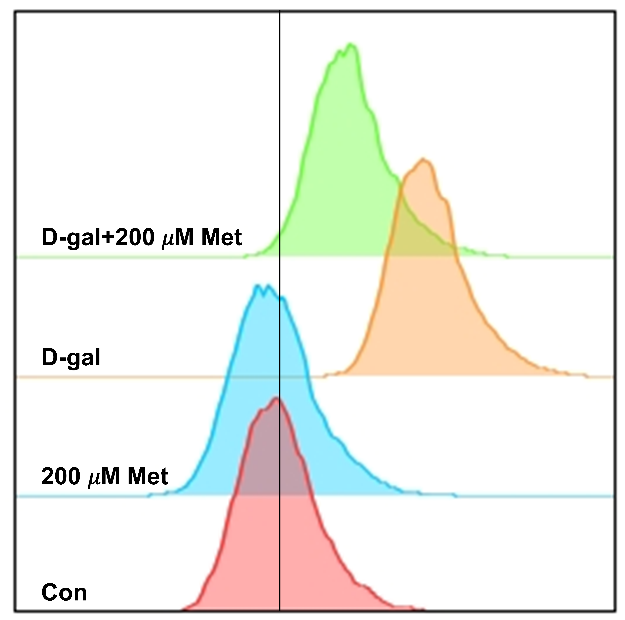


**Supplementary Figure S3**


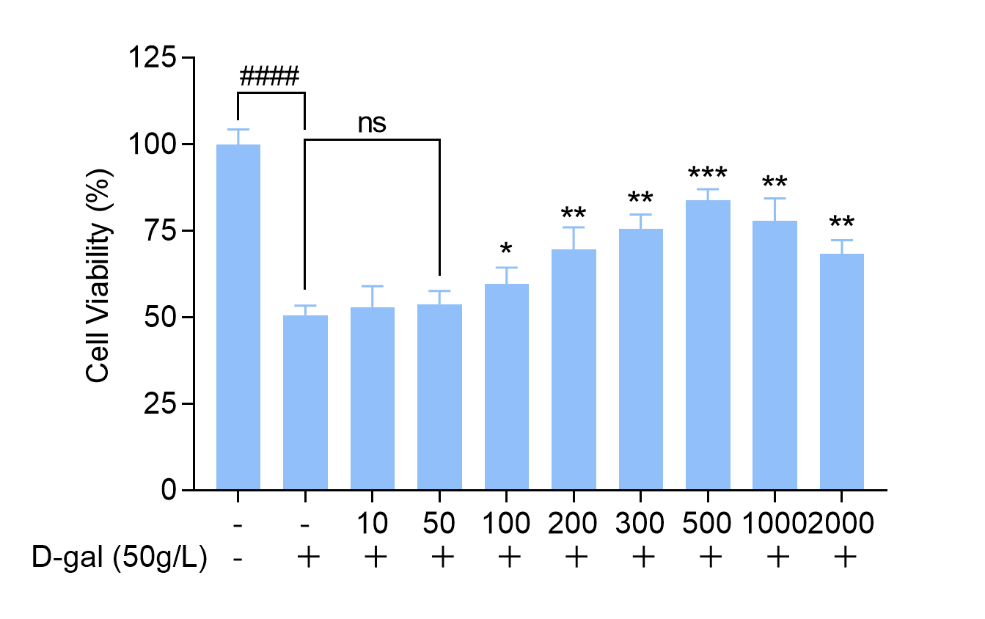


**Supplementary Figure S4**


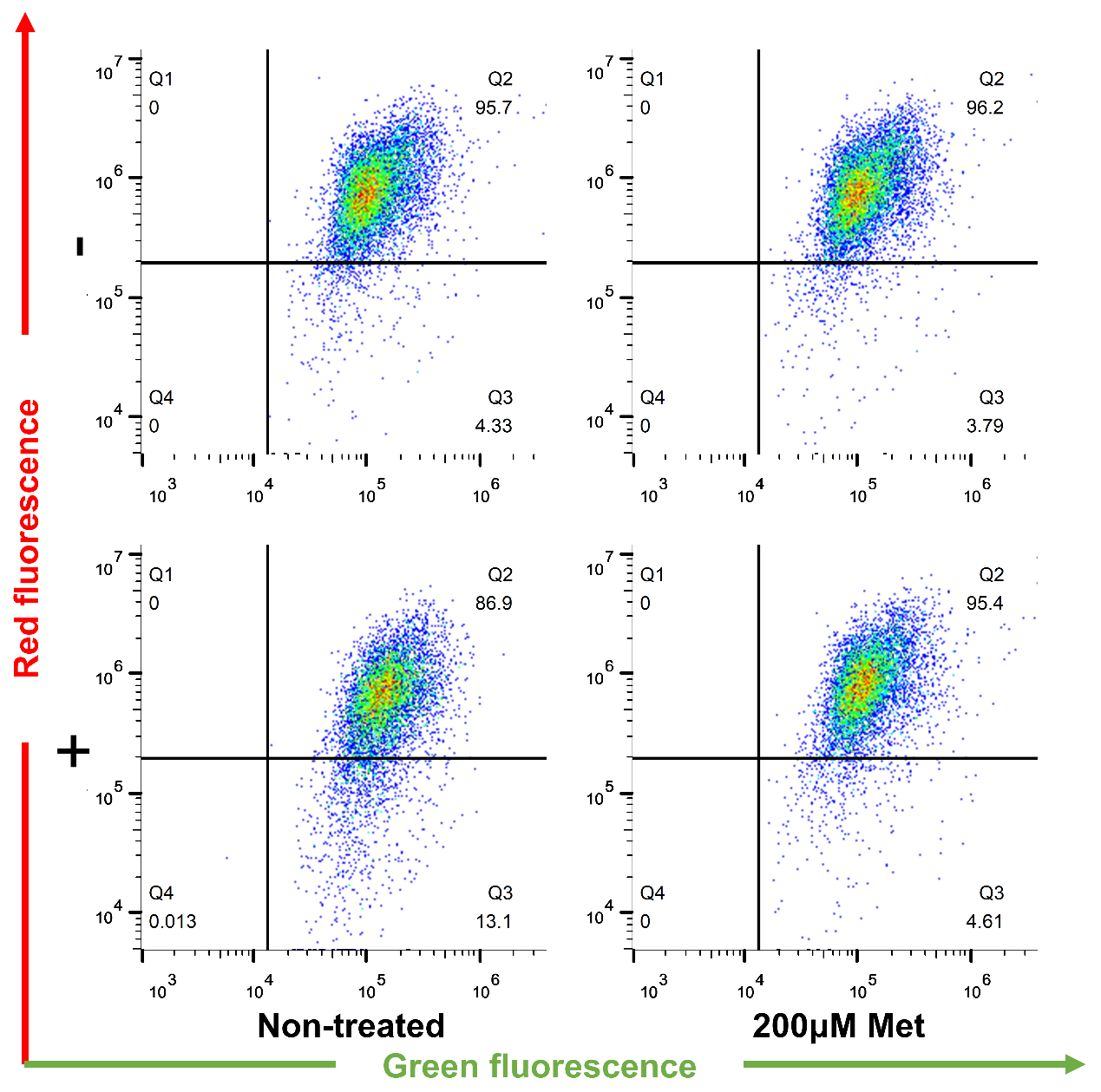

Supplement: Supplementary Materials — Supplementary Figure S1: the cell viability of hBMSCs was assessed by CCK8 assay following treatment with different concentrations of D-gal (0-100 g/L) for 12 h. n = 3 for each group. Data shown are mean ± SEM. Two-tailed t test. ∗p < 0.05, ∗∗∗p < 0.001, and ∗∗∗∗p < 0.0001 versus the control group; ns: not significant. Supplementary Figure S2: flow cytometry analysis of intracellular ROS levels. n = 3 for each group. Supplementary Figure S3: the cell viability of hBMSCs was assessed by CCK8 assay after being cultured for 72 h (at the concentration of 50 g/L D-gal). n = 3 for each group. Data shown are mean ± SEM. Two-tailed t test. ∗p < 0.05, ∗∗p < 0.01, and ∗∗∗p < 0.001 versus the D-gal group; ####p < 0.0001 versus the control group; ns: not significant. Supplementary Figure S4: flow cytometry analysis of MMP. n = 3 for each group. [file 1429642.f1.docx]
